# Brain-Targeted Nasal Clonazepam Microspheres

**Published:** 2009

**Authors:** J. Shaji, A. Poddar, S. Iyer

**Affiliations:** Prin. K. M. K. College of Pharmacy, Colaba, Mumbai-400 005, India

**Keywords:** Gelatin-chitosan cross linked microspheres, mucoadhesive microspheres, clonazepam, brain-targeted drug delivery, drug targeting index

## Abstract

Gelatin-chitosan mucoadhesive microspheres of clonazepam were prepared using the emulsion cross linking method. Mirospheres were evaluated using the *in vitro* and *ex vivo* drug release patterns. *In vivo* CNS drug distribution studies were carried out in rats by administering the clonazepam microspheres intra-nasally and clonazepam solution intravenously. From the drug levels in plasma and CSF, drug targeting index and drug targeting efficiency were calculated. Results obtained indicated that intranasally administered clonazepam microspheres resulted in higher brain levels with a drug targeting index of 2.12. Gelatin-chitosan cross linked mucoadhesive microspheres have the potential to be developed as a brain-targeted drug delivery system for clonazepam.

The nasal route opens up an avenue of possibilities for a drug to reach the brain. Clonazepam is highly metabolized with a poor uptake by the blood brain barrier via the peripheral circulation[1]. Hence, the objective of this investigation was to prepare brain-targeted gelatin-chitosan mucoadhesive microspheres to obtain rapid onset of action.

## MATERIALS AND METHODS

Clonazepam, USP was a gift sample from IPCA Laboratories, Mumbai; Chitosan was provided by Healer's Neutraceuticals, Chennai. All other chemicals and solvents were of analytical reagent grade and were locally procured. The emulsion cross-linking method was used[2]. Gelatin-chitosan mix along with clonazepam was added under constant stirring in paraffin oil. PEG 400 was the co-solvent employed. Glutaraldehyde-saturated toluene was used as the cross linking agent. Surfactant used was sodium lauryl sulfate (SLS, 0.01% v/v). After the microspheres were formed they were washed with isopropyl alcohol and air dried. The formulations prepared were showed in Table 1. Percent yield was determined by difference in weight before and after the preparation of microspheres[2].

**TABLE 1:** FORMULATION TABLE

Batch No.	Chitosan: gelatin ratio	Drug amount (mg)	Volume of SLS (0.01% v/v) (ml)	Aqueous to oily ratio
LM 1	2:5	50	1	1:20
LM 2	2:5	50	0.75	1:20
LM 3	2:5	50	0.5	1:20
LM 4	3:10	46.42	1	1:20
LM 5	3:10	46.42	0.75	1:20
LM 6	3:10	46.42	0.5	1:20
LM 7	2:10	45.85	1	1:20
LM 8	2:10	45.85	0.75	1:20
LM 9	2:10	45.85	0.5	1:20

**Fig. 1:**
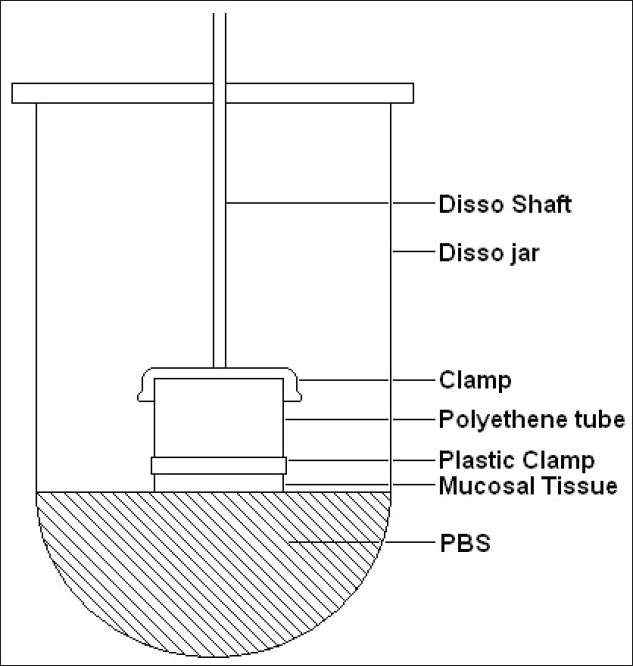
Modified dissolution apparatus Dissolution apparatus for evaluation of *ex vivo* permeation of clonazepam-loaded microspheres[6].

Particle size analysis was done microscopically using an optical microscope (Metzer OPTIK), under 450X (10X eye piece and 45X objective)[2]. One hundred particles were counted. Entrapment efficiency was calculated by soaking the microspheres in methanol and determining the content spectrophotometrically (V-550, Jasco, Japan)[3]. Evaluation of mucoadhesion was carried out using rat intestinal tissue. The amount of microspheres attaching to the tissue was determined by the weight difference[4]. For *in vitro* release, the paddle method (TDT-08L, Electrolab, India) of dissolution was employed over a period of 2 h. Release was compared against plain drug dissolution[5]. *Ex vivo* release was determined using sheep nasal mucosa[6]. Here too, the comparison was against plain drug solution. The set up used for this purpose is shown in fig. 1. Further, for *in vivo* studies, a comparison was made between the formulation administered via the nasal route and clonazepam solution administered intravenously to rats[7]. Plasma and CSF were withdrawn at set time points. Drug content was analyzed in both the fluids and concentration-time profiles were plotted and pharmacokinetics parameters calculated. Using these values, drug targeting index and drug targeting efficiency were found out.

## RESULTS AND DISCUSSION

**Fig. 2:**
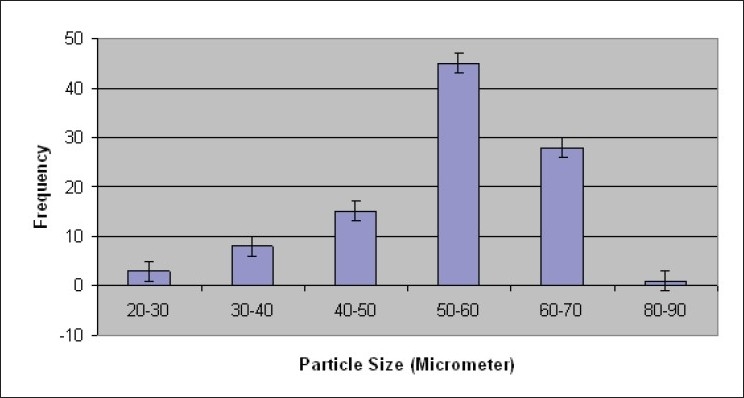
Size distribution for LM 1.

**Fig. 3:**
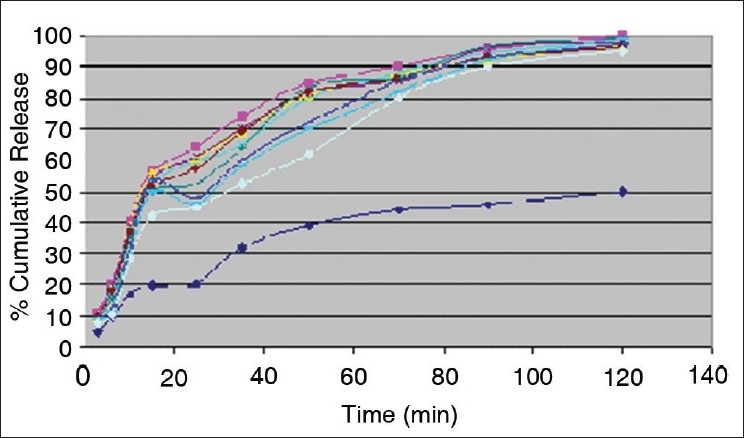
*In vitro* release studies of pure drug and the clonazepamloaded microspheres

**Fig. 4:**
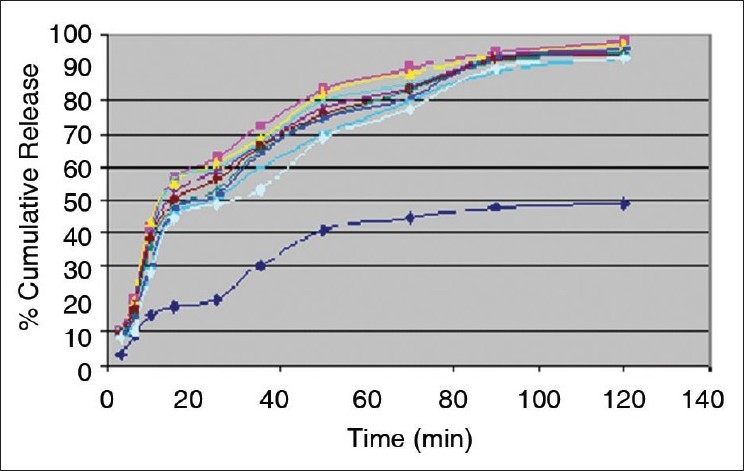
*Ex vivo* release studies of pure drug and the clonazepamloaded microspheres. *Ex vivo* release studies were performed using (
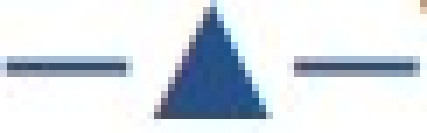
) pure drug and colazepam-loaded microspheres (
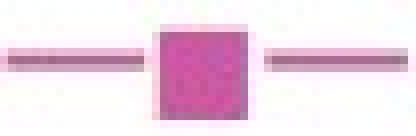
) LM1, (
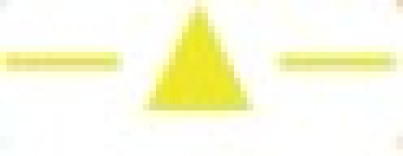
) LM2, (
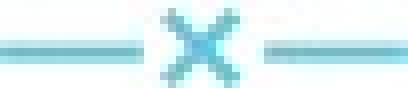
s) LM3, (
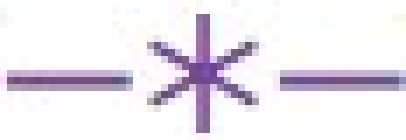
) LM4, (
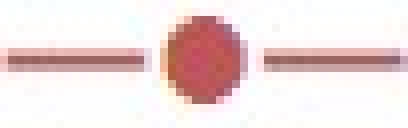
) LM5, (
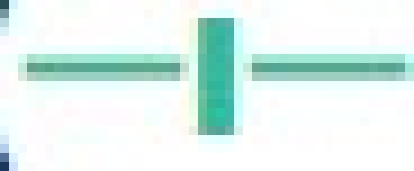
) LM6, (
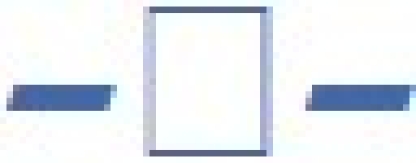
) LM7, (
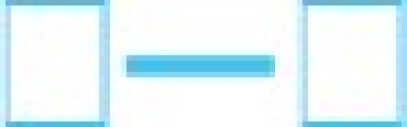
) LM8 and (
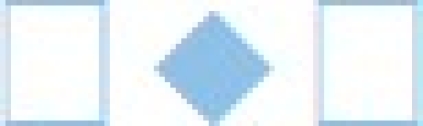
) LM9.

**Fig. 5:**
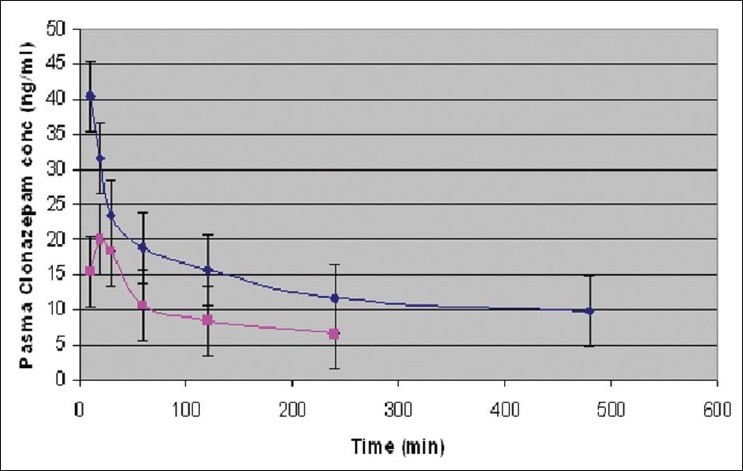
Plasma concentration-time profile of clonazpam after intravenous and intranasal administration Plasma concentration-time profile of clonazpam after (
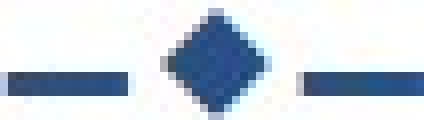
) intravenous and (
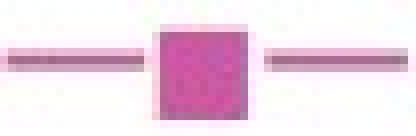
) intranasal administration of a 0.16-0.2 mg/kg dose in rats

**Fig. 6:**
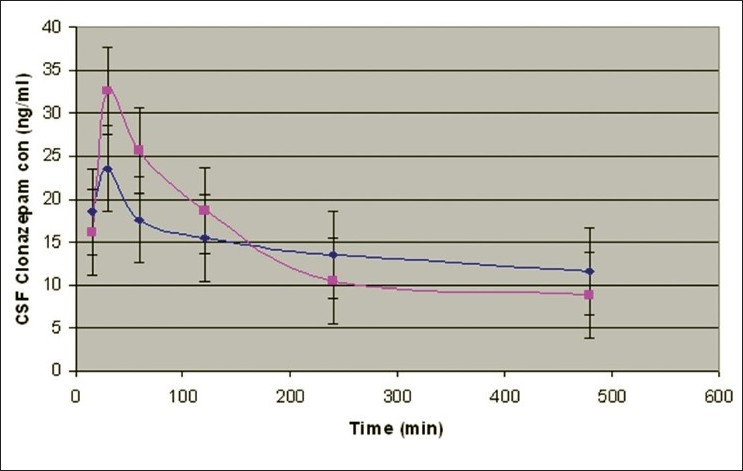
CSF concentration-time profile of clonazepam after intravenous and intranasal administration Cerebro spinal fluid concentration-time profile of clonazepam after (
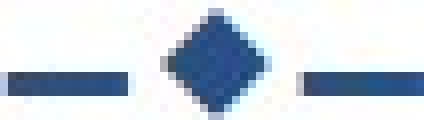
) intravenous and (
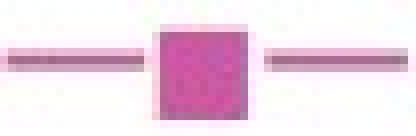
) intranasal administration of a 0.16-0.2 mg/kg dose in rats

Percent yield ranged from 65.1 to 75.4%. The average particle size ranged from 61-75.2 μm. Particle size distribution of LM 1 is shown in fig. 2. Volume of surfactant used played an important role in reducing the particle size. Entrapment values ranged from 38.9-54.23%. The use of PEG 400 enhanced entrapment. The mucoadhesion range was found to be 82.62-89.56%. Mucoadhesion value dropped with reducing the amount of chitosan. Results of the above parametes are tabulated in Table 2. The *in vitro* release obtained was 99.78% as seen in fig. 3. The *ex vivo* release achieved was 98.17 % as denoted in fig. 4. Chitosan crucially contributed towards a good release[6]. *In vivo* studies indicated higher concentration of clonazepam in CSF when administered intranasally as represented in figs. 5 and 6. Drug targeting index was 2.12 and drug targeting efficiency was 51.71%. Here again chitosan contributed towards a good release[6]. A drug targeting index value more than one indicates direct transport to the brain. Clonazepam is a low molecular weight (315.7) lipophilic moiety, thus indicating towards the possibility of utilizing the transcellular pathway to reach the brain[7]. Hence, a formulation was developed for better drug distribution into the brain, increasing the therapeutic index, reducing side effects with decreased dose for clonazepam.

*In vitro* release studies were performed using () pure drug and colazepam-loaded microspheres () LM1, () LM2, () LM3, () LM4, () LM5, () LM6, () LM7, () LM8 and () LM9.

**TABLE 2:** CHARACTRIZATIONS OF CLONAZEPAM LOADED MICROSPHERES

Batch no.	Percent yield	Average particle size (μm)*	Average entrapment efficiency (%)#	Average mucoadhesion (%)#
lM 1	65	61±1.88	54.23±0.5	89.56±0.4
LM 2	62	66.78±1.3	53.58±0.7	89.23±1.7
LM 3	74.2	75.2±0.66	51.29±2.1	88.7±2.2
LM 4	75.1	69.45±0.71	48.83±1.8	86.38±1.2
LM 5	63.89	69.3±1.54	43.41±0.9	85.92±0.8
LM 6	61.74	72±0.94	44.6±0.6	84.81±1.5
LM 7	70.16	73.6±0.45	42.5±0.4	85.13±1.8
LM 8	64.04	74.4±0.81	41.76±1.3	83.8±2.2
LM 9	71.3	74.2±0.64	38.9±1.5	82.61± 0.5

* Values expressed as mean±SD, n=100

# Values expressed as mean±SD, n=3
